# Personal

**Published:** 1910-12

**Authors:** 


### PERSONAL

---

DR. H. C. ROOTH, of Buffalo, who has been abroad for three months, doing special surgical work at Vienna and London, has returned and resumed his professional work.

Dr. William H. Hodge, of Niagara Falls, has been appointed by Governor White as manager of the Western House of Refuge at Albion.

Dr. William B. May, milk inspector in the Buffalo health department, has been appointed chief of the division of communicable diseases in the state department of health to succeed Dr. William A. Howe, of Phelps, who has been made deputy state commissioner of health.

Dr. Eugene A. Smith, one of the attending surgeons of the Sisters' of Charity Hospital, has returned to Buffalo after an absence of several months. He received a warm welcome at the hospital and from his many friends in the city.

Dr. James Francis Rice, of 551 Elmwood Avenue, Buffalo, opened on October 1, 1910, another office, the second being at 164 East North Street, corner of Michigan. After the first of the year, when he gives up his present position as Medical Referee of the Mutual Life Insurance Company of New York, he will keep two office hours at both offices—namely, at Elmwood Avenue and at North Street. At present he is at the new office only from 6 to 7 p. m., and by appointment.

